# Buffy Coat Enrichment Improves the Success Rate of Conventional Cytogenetics in Hypocellular Specimens: A Prospective Quality Improvement Study

**DOI:** 10.3390/genes17040417

**Published:** 2026-03-31

**Authors:** Gokce A. Toruner, Nawal Imtiaz, Andrew M. McCoy, Melissa Robinson, Griselda J. Cardona, Sarita S. Delgado, Su Yang, Qing Wei, L. Jeffrey Medeiros, Guilin Tang

**Affiliations:** Department of Hematopathology, The University of Texas MD Anderson Cancer Center, Houston, TX 77030, USAljmedeiros@mdanderson.org (L.J.M.);

**Keywords:** chromosomal analysis, karyotyping, hypocellular, buffy coat enrichment, quality improvement

## Abstract

**Background**: Specimens with low cell counts may fail to yield sufficient analyzable metaphases or are rejected for chromosomal analysis. Buffy coat enrichment (BCE) concentrates nucleated cells prior to culture; however, its impact on routine cancer cytogenetics has not been systematically evaluated. **Methods**: We first validated BCE in hypocellular specimens (<5 K/µL) and then conducted a prospective quality improvement study from November 2024 to October 2025, encompassing 12,088 specimens. A phased intervention strategy was implemented by performing BCE on specimens with cell counts of 2.0–4.9 K/µL (designated as phase I); followed by expanding BCE to specimens with cell counts of 1.0–4.9 K/µL (phase II). Outcomes were assessed by the rate of successful karyotypes, defined as ≥10 analyzable metaphases. **Results**: In the validation cohort (cell counts < 5 K/µL), BCE improved the success rate across all cell count strata. In the prospective study cohort, implementation of BCE increased the overall success rate from 78% at baseline to 83% in phase I, and further increased to 90% in phase II. **Conclusions**: BCE significantly improves the success rate of chromosomal analysis by increasing the yield of metaphases in hypocellular specimens. This simple and scalable intervention reduces specimen rejection and enhances diagnostic yield in routine cancer cytogenetics.

## 1. Introduction

Chromosome analysis plays a critical role in the diagnosis, classification, and risk stratification of hematologic malignancies, thereby enhancing therapeutic decision-making for patients [1,2,3,4]. In addition to guiding patient management, cytogenetic data are often required by clinical trial sponsors, underscoring the importance of these data in both clinical and research settings.

Successful chromosome analysis depends on obtaining high-quality metaphases by G-banding, which in turn requires robust cell culture. Several factors influence culture success, and one of the most important factors is the cell count. The “cell” here refers to all nucleated cells in bone marrow (BM) and peripheral blood (PB), or white blood cells (WBCs) in PB. Other factors that may affect the metaphase yield include culture media, culture time and conditions, and the use of mitogens [5]. For the conventional cytogenetic workup of hematologic malignancies, BM and PB are the most common specimen types. The optimal cell concentration in culture is typically 1–2 × 10^6^ cells/mL (equivalent to 1–2 K/µL). A standard 10 mL culture therefore corresponds to a total of 10–20 million cells. To achieve this cellularity, laboratories generally inoculate approximately 0.25–1.0 mL of BM aspirate or PB into culture to make a 10 mL culture. Samples containing fewer than 5 million cells are often considered suboptimal for standard cytogenetic analysis [6]. When a specimen is deemed unlikely to yield adequate metaphases (e.g., markedly hypocellular samples), it is often rejected prior to culture setup to avoid unnecessary consumption of laboratory resources. However, such rejections preclude the generation of critical cytogenetic information and may adversely affect patient care.

The term “buffy coat” was first used in 1819 [7], and its characteristics were further described in 1962 [8]. The technique for separating blood components by density gradient centrifugation was developed in 1974 [9]. Briefly, the buffy coat is a white or yellow-white layer that forms between the plasma (top layer) and the red blood cells (RBCs, bottom layer) after centrifugation. It is highly concentrated with nucleated cells (granulocytes, lymphocytes, monocytes, erythroid precursors, plasma cells, leukemia and/or lymphoma cells, etc.) and platelets. These nucleated cells are the primary source of metaphases required for chromosome analysis. Buffy coat preparation has been used to enrich nucleated cells for various genetic applications [10,11,12,13,14,15,16,17], including cytogenetic testing [18,19,20,21].

In this study, we evaluated the impact of buffy coat enrichment (BCE) on chromosomal analysis in specimens with low nucleated cell counts (<5 K/µL) processed in our laboratory. We hypothesized that BCE would improve culture success and increase the proportion of reportable karyotypes in hypocellular samples.

## 2. Materials and Methods

### 2.1. Study Design and Specimens

This study included a retrospective validation cohort and a prospective cohort. The validation cohort comprised 365 hypocellular specimens, of which 306 did not undergo buffy coat enrichment (BCE) prior to tissue culture and 59 received BCE. Based on favorable performance of BCE in hypocellular specimens, a prospective study was conducted including 12,088 patients between November 2024 and October 2025, during which three sample rejection criteria were evaluated. Under baseline criteria (November 2024–February 2025), specimens with cell counts <3.0 K/µL were rejected and BCE was performed for counts of 3.0–4.9 K/µL; during Intervention I (March–June 2025), the rejection threshold was lowered to <2.0 K/µL with BCE applied to specimens with counts of 2.0–4.9 K/µL; and during Intervention II (July–October 2025), specimens with counts <1.0 K/µL were rejected and BCE was performed for counts of 1.0–4.9 K/µL

### 2.2. Specimen Receipt and Culture Setup for Chromosome Analysis

BM aspirate and PB for chromosome analysis were collected in sodium heparin tubes. Upon receipt in the Cytogenetics Laboratory, each specimen was accessioned and transferred into a sterile transport vial which was then securely capped. A cell count was obtained using th hematology analyzer (Symex, Kobe, Japan), and both the cell count and specimen volume were recorded in the laboratory running log.

Chang Marrow or Marrow Max media were used for all culture setups. Cultures were established as a whole culture (10 mL) or a half culture (5 mL), inclusive of specimen and media, and incubated for 24, 48, or 72 h. The 72 h cultures contained one of the following mitogens based on clinical indications: CpG–IL2 for B-cell leukemia/lymphoma and myeloma, phytohemagglutinin (PHA) for T-cell neoplasms, or pokeweed mitogen (PPC) for chronic lymphocytic leukemia (CLL).

### 2.3. Buffy Coat Enrichment

When a specimen had a nucleated cell count of between 1.0 and 4.9 K/µL, it was centrifuged at 1200 rpm for 7 min. The buffy coat layer was carefully aspirated using a plastic pipette with a squeeze bulb, avoiding contamination from the plasma or red blood cell layers. The isolated buffy coat was then inoculated into culture medium to a final volume of 5 mL.

### 2.4. Categories of Chromosomal Analysis

Five categories were designated. For comparison and analysis purposes, we combined categories #1 and #2 into “No cytogenetic results” or “failure”, and combined categories #4 and #5 into “Successful karyotyping”.

1. Specimen rejected: specimens with a cell count below the acceptance threshold (see section on phases).
2. No mitotic cells (NMC): cultures were set up, but no analyzable metaphase cells were observed.
3. Insufficient: 1–9 metaphases analyzed.
4. Incomplete: 10–19 metaphases analyzed.
5. Complete: 20 metaphases analyzed.

### 2.5. Data Analysis

Data analysis was performed by Minitab 13.0.

## 3. Results

### 3.1. Validation Cohort

We compared successful karyotyping rates (≥10 analyzable metaphases) among hypocellular specimens processed with and without BCE.

**Figure 1:**
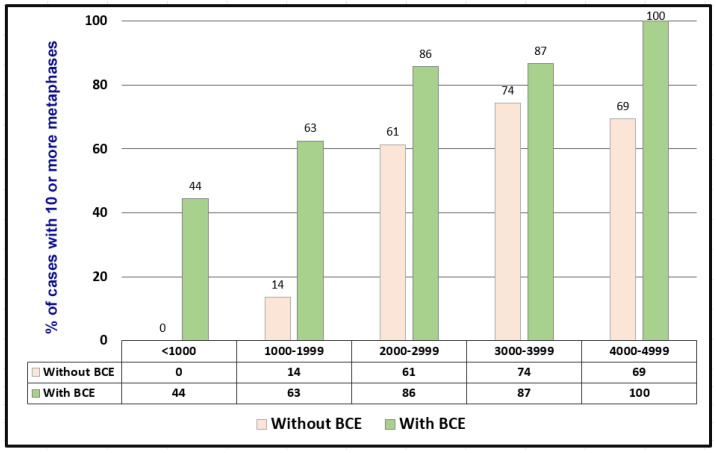
Validation of buffy coat enrichment (BCE) for chromosomal analysis. Comparison of the success rate (≥10 analyzable metaphases) among specimens with cell counts <5 K/µL, processed with or without BCE. BCE significantly improved success rates across all cell count categories.

Cultures without BCE: A total of 306 specimens (302 BM and 4 PB) with cell counts <5 K/µL were inoculated into half cultures without BCE. The distribution of cell counts and cytogenetic outcomes is summarized in Table 1. The proportions of cases yielding 10–20 analyzable metaphases were 0%, 14%, 61%, 74%, and 69% for specimens with cell counts of <1.0, 1.0–1.9, 2.0–2.9, 3.0–3.9, and 4.0–4.9 K/µL, respectively (Table 1, Figure 1)

#### Cultures with BCE

A total of 59 BM aspirates with cell counts <5 K/µL underwent BCE prior to culture. The proportions of cases yielding 10–20 analyzable metaphases were 44%, 63%, 86%, 87%, and 100% for specimens with cell counts of <1.0, 1.0 (–,1.9, 2.0 (–,2.9, 3.0 (–,3.9, and 4.0 (–,4.9 K/µL, respectively (Figure 1).

Across all cell count categories, specimens processed with BCE demonstrated consistently higher successful karyotyping rates compared with those cultured without BCE.

### 3.2. Prospective Study Cohort (November 2024–October 2025)

#### 3.2.1. Specimens and Cell Counts

Between 1 November 2024 and 31 October 2025, 12,088 specimens were processed in our clinical cytogenetics laboratory: 94% were BM aspirates, 5% were PB, and the remaining 1% were fine needle aspiration (FNA) or tissue biopsy specimens.

During the 12-month study period, 68% of specimens (range, 65–72%) had a cell count >5 K/µL; 14% (range, 13–16%) had a cell count of 3.0–4.9 K/µL; 7% (range, 5–8%) had a cell count of 2.0–2.9 K/µL; 7% (range, 6–9%) had a cell count of 1.0–1.9 K/µL; and 5% (range, 3–6%) had a cell count of 0–0.9 K/µL (Table 2). Case-level data is presented in Supplementary Table S1.

#### 3.2.2. Baseline Phase (1 November 2024–28 February 2025)

Specimens with cell counts <3.0 K/µL were rejected for chromosome analysis. BCE was performed on specimens with cell counts of 3~4.9 K/uL.

**Figure 2:**
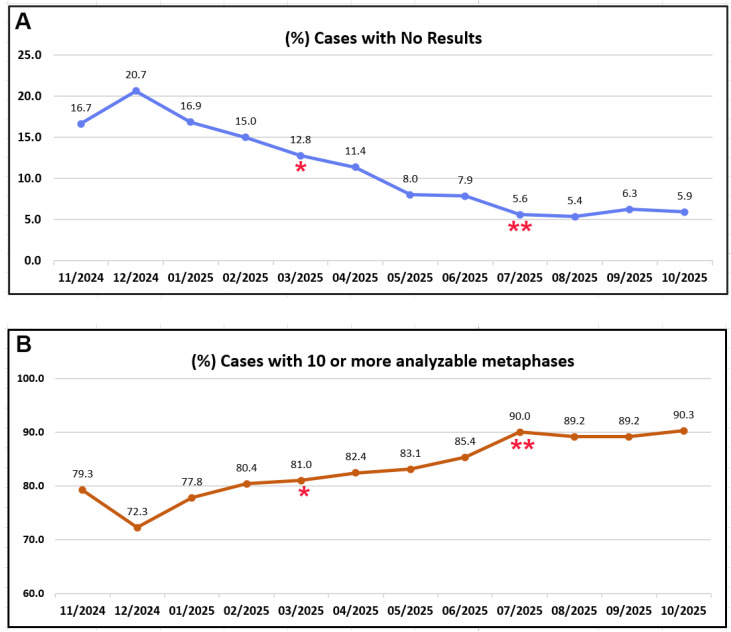
(**A**) Failure rate (no cytogenetic result) and (**B**) success rate (≥10 analyzable metaphases) among all 12,088 specimens received over a 12-month period. * Intervention I implemented; ** Intervention II implemented.

The outcomes of chromosome analysis are summarized in Table 3 and Figure 2. During the baseline period, 13–19% of specimens were rejected. An additional 1–2% of cases yielded no mitotic cells (NMC). The overall failure rate (rejection plus NMC) ranged from 15% to 21%. The successful karyotyping rate ranged from 72% to 80% during these four months.

#### 3.2.3. Phase I (1 March 2025–30 June 2025)

During phase I, the rejection threshold was lowered: specimens with cell counts <2.0 K/µL were rejected, and BCE was performed for specimens with cell counts of 2.0–4.9 K/µL. As shown in Table 3, 4–11% of specimens were rejected, and 2–4% yielded NMC, resulting in an overall failure rate of 8–13%. The successful karyotyping rate increased to 81–85% (Figure 2).

**Figure 3:**
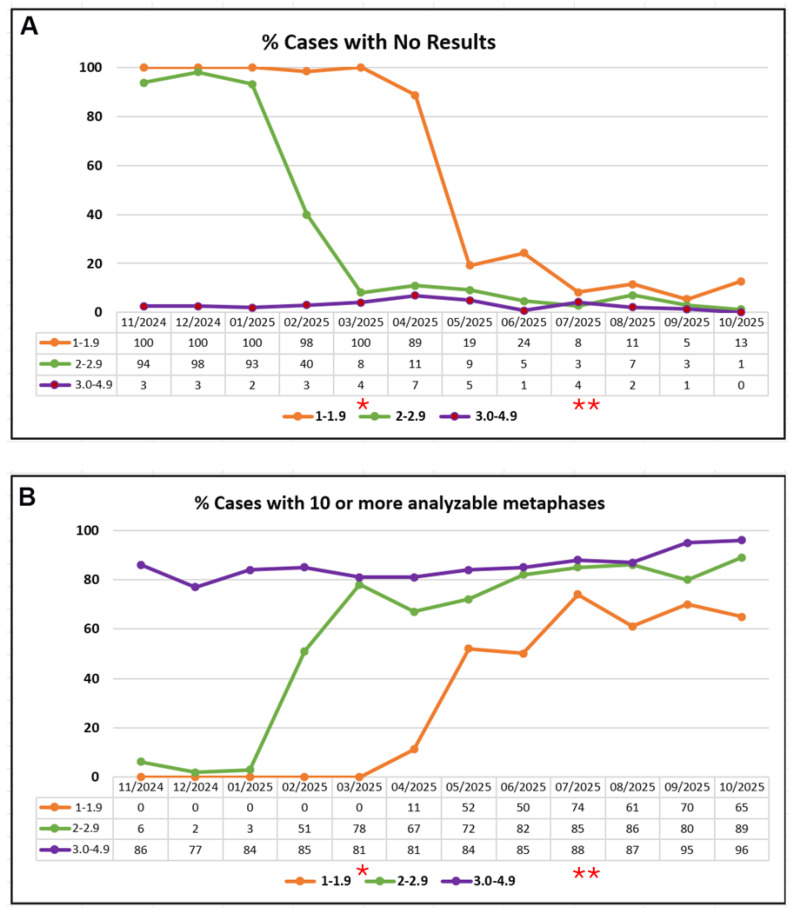
(**A**) Failure rate (no cytogenetic result) and (**B**) success rate (≥10 analyzable metaphases) among hypocellular specimens received over a 12-month period, stratified by cell count groups of 1.0 –1.9 K/µL, 2.0 –2.9 K/µL, and 3.0 –4.9 K/µL. * Intervention I implemented; ** Intervention II implemented.

Within the 2.0–2.9 K/µL subset, 5–11% of cases had no cytogenetic results due to rejection or NMC (Figure 3A). The successful karyotyping rate in this group improved to 67–82% following implementation of BCE (Figure 3B). These improvements were sustained in subsequent months.

#### 3.2.4. Phase II (1 July 2025–31 October 2025)

During phase II, the rejection threshold was further lowered: specimens with cell counts <1.0 K/µL were rejected, and BCE was performed for specimens with cell counts of 1.0–4.9 K/µL. As summarized in Table 3, 3–5% of specimens were rejected, and approximately 2% yielded no mitotic cells, resulting in an overall failure rate of 6–7%. The successful karyotyping rate increased further to 89–90% (Figure 2).

Within the 1.0–1.9 K/µL subset, 5–13% of cases had no cytogenetic results due to rejection or NMC (Figure 3A). In contrast, the successful karyotyping rate improved to 61–74% after implementation of BCE (Figure 3B). These performance metrics remained stable during the study interval and in later routine practice.

## 4. Discussion

Successful cytogenetic analysis depends on adequate specimen cellularity and volume to support reliable culture growth and metaphase generation. In routine practice, laboratories must balance maximizing diagnostic yield with responsible use of technologist time, culture capacity, and laboratory resources. Historically, specimens with low cell counts (<3 K/µL) have often been rejected because of the anticipated high likelihood of culture failure. However, such rejection eliminates the opportunity to generate clinically actionable cytogenetic information.

In this prospective quality improvement study encompassing more than 12,000 specimens, we demonstrate that buffy coat enrichment (BCE) substantially improves cytogenetic success in hypocellular samples. The validation cohort confirmed that BCE increases the likelihood of achieving ≥10 analyzable metaphases across all cell count strata below 5 K/µL.

A central concern when lowering acceptance thresholds is the possibility of converting previously rejected specimens into cultures that ultimately yield no mitotic cells, thereby consuming resources without improving diagnostic output. Our phased implementation strategy directly addressed this issue. Following phase I, in which BCE was applied to specimens with cell counts of 2.0–2.9 K/µL, the overall failure rate declined substantially without a disproportionate increase in cases with no mitotic cells (NMC). Expansion of BCE to the 1.0–1.9 K/µL cell count group during phase II further reduced overall failure rates while maintaining stable NMC proportions. These findings indicate that BCE does not simply redistribute failure categories; rather, it meaningfully increases the probability of generating analyzable metaphases and reportable karyotypes.

Importantly, specimens with cell counts between 1.0 and 2.9 K/µL accounted for approximately 14% of all submissions during the study period. By implementing BCE in this subgroup, the laboratory was able to convert a substantial number of previously rejected or unsuccessful cases into successful cytogenetic studies, thereby increasing overall diagnostic output.

This study has several strengths, including prospective implementation, continuous monthly performance monitoring, and a large sample size reflective of real-world clinical practice. Nonetheless, limitations should be acknowledged. The study was conducted at a single institution, and pre-analytic handling, specimen mix, and culture practices may vary across laboratories. In addition, although we evaluated success based on metaphase yield, we did not formally assess banding resolution or sensitivity for structural abnormality detection. While routine case review did not reveal diminished banding quality after BCE, systematic evaluation of karyotype quality metrics would strengthen future analyses. A formal cost-effectiveness assessment was also not performed; however, BCE requires minimal additional equipment or consumables beyond standard centrifugation, suggesting that implementation costs are low.

In summary, buffy coat enrichment represents a practical and scalable strategy that enables laboratories to safely broaden acceptance criteria for hypocellular specimens. By reducing rejection rates and increasing the proportion of cases with complete cytogenetic analysis, BCE enhances diagnostic yield without compromising culture performance. This approach may be readily adopted in other high-volume cytogenetics laboratories seeking to optimize operational efficiency and improve patient care.

## Figures and Tables

**Table 1:** Outcomes of chromosomal analysis of hypocellular specimens with cell counts <5 K/µL. No buffy coat enrichment performed on these specimens. NMC: no metaphase cells.

	Cell Count/µL	<1000	1000–1999	2000–2999	3000–3999	4000–4999	Total
Metaphase							
NMC		6 (22)	21 (36)	10 (13)	3 (4)	6 (8)	46 (15)
<10 cells		21 (78)	30 (51)	19 (25)	15 (21)	17 (23)	102 (33)
10~19 cells		0 (0)	8 (14)	12 (16)	13 (19)	1 (1)	34 (11)
20 cells		0 (0)	0 (0)	34 (45)	39 (56)	51 (68)	124 (41)
Total Case Number		27	59	75	70	75	306

Data presented: case number (%).

**Table 2:** Cell count distribution of all cases received during a 12-month period. Cases are grouped based on cell counts.

	Month	Nov. 2024	Dec. 2024	Jan. 2025	Feb. 2025	Mar. 2025	Apr. 2025	May. 2025	Jun. 2025	Jul. 2025	Aug. 2025	Sep. 2025	Oct. 2025	All Cases
Cell Count K/uL														
0–0.9		43 (5)	51 (6)	47 (4)	49 (5)	48 (5)	37 (4)	47 (5)	55 (5)	46 (4)	33 (3)	48 (5)	50 (5)	554 (5)
1–1.9		54 (6)	71 (8)	75 (7)	64 (6)	60 (6)	62 (6)	89 (9)	66 (7)	84 (8)	87 (9)	73 (7)	71 (6)	856 (7)
2–2.9		48 (5)	53 (6)	58 (5)	80 (8)	74 (8)	82 (8)	77 (8)	65 (6)	74 (7)	71 (7)	71 (7)	81 (7)	834 (7)
3–4.9		118 (13)	115 (13)	149 (14)	162 (16)	124 (13)	129 (13)	141 (14)	135 (13)	142 (13)	139 (14)	149 (15)	161(15)	1664 (14)
>5		662 (72)	625 (68)	775 (70)	679 (66)	680 (69)	675 (69)	654 (65)	683 (68)	708 (67)	658 (67)	649 (66)	732 (67)	8180 (68)
Total case number		925	915	1104	1034	986	985	1008	1004	1054	988	990	1095	12,088

Data presented: case number (%).

**Table 3:** Outcomes of chromosomal analysis of all specimens received during a 12-month period. Baseline: Specimens with cell counts <3.0 K/µL were rejected for chromosome analysis. Buffy coat enrichment (BCE) was performed on specimens with cell counts of 3~4.9 K/µL. Intervention I: specimens with cell counts <2.0 K/µL were rejected. BCE was performed for specimens with cell counts of 2~4.9 K/µL. Intervention II: specimens with cell counts <1.0 K/µL were rejected. BCE was performed for specimens with cell counts of 1~4.9 K/µL. NMC: no metaphase cells.

		Baseline				Intervention I				Intervention II				
	Month	Nov. 2024	Dec. 2024	Jan. 2025	Feb. 2025	Mar. 2025	Apr. 2025	May. 2025	Jun. 2025	Jul. 2025	Aug. 2025	Sep. 2025	Oct. 2025	All Cases
Metaphase														
Rejected		143 (15)	173 (19)	175 (16)	137 (13)	107 (11)	91 (9)	43 (4)	53 (5)	40 (4)	31 (3)	47 (5)	48 (4)	1088 (9)
NMC		11 (2)	16 (2)	11 (1)	18 (2)	19 (2)	21 (2)	38 (4)	26 (3)	19 (2)	22 (2)	15 (2)	17 (2)	233 (2)
1–9 met		38 (4)	65 (7)	59 (5)	48 (5)	61 (6)	61 (6)	89 (9)	68 (7)	46 (4)	54 (5)	45 (5)	42 (4)	676 (6)
10–19 met		28 (3)	35 (4)	44 (4)	46 (4)	49 (5)	51 (5)	60 (6)	55 (5)	58 (6)	45 (5)	38 (4)	52 (5)	561 (5)
20 met		705 (76)	626 (68)	815 (74)	785 (76)	750 (76)	761 (77)	778 (77)	802 (80)	891 (85)	836 (85)	845 (85)	936 (85)	9530 (79)
Total Case No		925	915	1104	1034	986	985	1008	1004	1054	988	990	1095	12,088

## Data Availability

Data are contained within the article.

## Supplementary Materials

The following supporting information can be downloaded at https://www.mdpi.com/article/10.3390/genes17040417/s1, Table S1: Case Level Data.
